# Effectiveness of post-exposition prophylaxis with oseltamivir in nursing homes: a randomised controlled trial over four seasons

**DOI:** 10.1186/1742-7622-11-13

**Published:** 2014-09-10

**Authors:** Marianne AB van der Sande, Adam Meijer, Fatmagül Şen-Kerpiclik, Remko Enserink, Herman JM Cools, Piet Overduin, José M Ferreira, Marie-José Veldman-Ariessen

**Affiliations:** 1 Centre Infectious Disease Control, RIVM, PO Box 1-pb75, 3720BA, Bilthoven, the Netherlands; 2 Julius Centre for Health Sciences and Primary Care, UMC Utrecht, Utrecht, Netherlands; 3 Department Public Health and Primary Care, Leiden University Medical Centre, Leiden, Netherlands; 4 RIO, RIVM, Bilthoven, Netherlands

**Keywords:** Influenza outbreaks, Post-exposition prophylaxis, Nursing homes, RCT, Oseltamivir

## Abstract

**Background:**

Oseltamivir has been registered for use as post-exposition prophylaxis (PEP) following exposure to influenza, based on studies among healthy adults. Effectiveness among frail elderly nursing home populations still needs to be properly assessed.

**Methods:**

We conducted a randomised double-blind placebo-controlled trial of PEP with either oseltamivir (75 mg once daily) or placebo among nursing home units where influenza virus was detected; analysis was unblinded. The primary outcome was laboratory-confirmed influenza among residents in units on PEP; the secondary outcome was clinical diagnosis of influenza-like illness (ILI).

**Results:**

42 nursing homes were recruited, in which 17 outbreaks occurred from 2009 through 2013, two caused by influenza virus B, the others caused by influenza virus A(H3N2). Randomisation was successful in 15 outbreaks, with a few chance differences in baseline indicators. Six outbreaks were assigned to oseltamivir and nine to placebo. Influenza virus positive secondary ILI cases were detected in 2/6 and 2/9 units respectively (ns); secondary ILI cases occurred in 2/6 units on oseltamivir, and 5/9 units on placebo (ns). Logistical challenges in ensuring timely administration were considerable.

**Conclusion:**

We did not find statistical evidence that PEP with oseltamivir given to nursing home residents in routine operational settings exposed to influenza reduced the risk of new influenza infections within a unit nor that of developing ILI. Power however was limited due to far fewer outbreaks in nursing homes than expected since the 2009 pandemic. (RCT nr NL92738)

## Background

Nursing home residents belong to the most vulnerable groups as far as risk of serious influenza-related morbidity and mortality is concerned. Therefore, nearly all nursing home residents receive annual influenza vaccinations. However, in this population the immune response to vaccination can be reduced related to age and co-morbidity, and routine vaccination may not be sufficiently protective for many vaccinated residents in relation to dose and boosting, especially in those with low pre-vaccination titers [1-5]. A reduced immune response could also induce serious, protracted illness, as well as slow recovery. Prevention of influenza outbreaks in nursing homes could therefore have major health gains for the residents as well as reduce excess burden for nursing homes.

Oseltamivir can inhibit replication of influenza A and B viruses, and may prevent further human-to-human transmission [6]. Experimental [7] and observational [8] studies among healthy adults and trials among healthy mainly unvaccinated household contacts [9,10] showed high effectiveness of prophylactic oseltamivir in prevention of transmission, even when the index case was not treated [10]. These observations cannot be extrapolated directly to nursing home residents, where a high vaccination coverage might affect viral replication and excretion; a high level of co-morbidity may affect relevant cellular and immunological responses, multiple co-medication could lead to drug interactions, and aging may modulate the regular immune response. Nevertheless, observational studies in nursing homes [11-14], also in the Netherlands [15], have suggested that also here PEP with oseltamivir could be effective in preventing transmission. So far, only one randomised multi-centre study on the effectiveness of oseltamivir prophylaxis among nursing home residents has been published. Here, oseltamivir was prescribed prophylactically for 6 weeks and not as PEP, but a 92% reduction in the incidence of laboratory confirmed influenza compared with placebo was noted [16].

The uncertainty about the evidence-base for effectiveness of PEP with oseltamivir in nursing homes is reflected in the literature [17-24]. While acknowledging the absence of sufficient data, both the Dutch Association of Nursing Homes [25] and the Dutch National Centre for Infectious Disease Control [26] recommended in 2004 implementation of PEP with oseltamivir in nursing home units with a laboratory confirmed influenza patient, but also recommended that if PEP was used, it should be scientifically evaluated. A similar recommendation has been formulated in the UK [27]. An evaluation in the Netherlands at the end of the 2004–2005 season showed that although 65% of nursing homes with a confirmed influenza outbreak had implemented PEP at least to some degree, there was no consensus on this policy. In particular the lack of evidence for effectiveness in nursing homes was mentioned (by 34%) as a major concern [28].

In view of the high risk among nursing home residents of medical complications following an influenza infection, persistent low uptake of influenza vaccination among nursing home staf [29], insufficient evidence from RCTs in this specific setting, and the potentially considerable budgetary, ethical and logistical impact of the proposed intervention, we conducted a double-blind placebo-controlled randomised controlled trial to assess the effectiveness of PEP with oseltamivir on prevention of influenza infection in this environment.

## Methods

### Trial design

This was a double-blind, placebo-controlled randomised controlled trial among Dutch nursing homes during four influenza seasons in the period 2009–2013, coordinated by the Centre for Infectious Disease Control (CIb) in the Dutch National Institute for Public Health (RIVM). We assumed that following a virological diagnosis of influenza, without oseltamivir further transmission would occur in 40% of units, and we assumed that PEP with oseltamivir will result in a reduction of at least 70% in units with new symptomatic influenza-confirmed cases [9]. Then, we calculated we would need to randomise 60 units to demonstrate such a reduction with at least 80% power at the 5% significance level. Assuming 30% of nursing homes experience an influenza outbreak, on average in 2 units, we planned the study to run over three seasons. Due to the expected unexpected pandemic, resulting in much lower attack rates among the elderly, and very few outbreaks in nursing homes since, the study was extended to four seasons.

Nursing homes were recruited throughout the country, with a view to obtaining a representative sample of Dutch nursing homes. After written consent was obtained from the nursing home, residents or if indicated their legal representatives were provided with information and requested for their written consent to participate in the trial, in case their unit would be hit by influenza and be randomised to PEP with study medication. As turn-over of residents in nursing homes is considerable, this procedure to inform and obtain consent before any actual outbreak was updated every year in the autumn, prior to the start of the influenza season. Furthermore, when an actual outbreak occurred, all residents on the affected unit who had not previously consented or declined to participate, were still requested for their consent to participate. Within each unit with an influenza outbreak, all consenting residents who were not ill were eligible for PEP. Units were excluded if there were less than five consenting residents; individual residents diagnosed by their treating physicians to suffer from chronic renal failure were not eligible [30,31].

In each participating nursing home, a contact person was identified who was responsible for coordinating and managing the study. All participating nursing homes were visited to explain the study to staff and management; staff were trained in identification of ILI and standardised collection of respiratory specimens and data. ILI was clinically diagnosed by the treating physicians in the nursing homes, and defined according to the ECDC EU case definition as a sudden onset of symptoms and at least one of the following four systemic symptoms: fever or feverishness, malaise, headache, myalgia, and at least one of the following three respiratory symptoms: cough, sore throat, shortness of breath (http://ecdc.europa.eu/en/activities/surveillance/eisn/surveillance/pages/influenza_case_definitions.aspx). An influenza outbreak was defined as virological confirmation of influenza in a respiratory specimen of one or more residents with ILI from the same unit where ILI was diagnosed. For each nursing home, the collaborating laboratory was identified and informed about the study by the study team.

**Figure 1:**
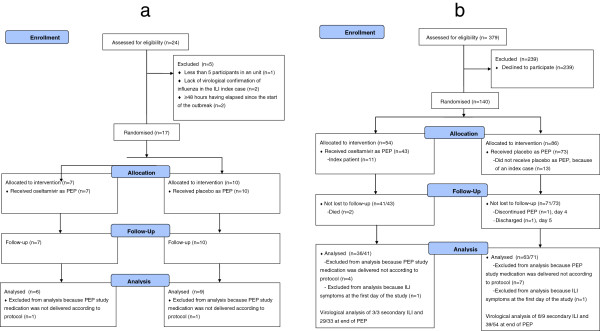
Flow diagram a) by outbreak; b) by participants.

In case of a suspected influenza outbreak in a nursing home unit, a nasopharyngeal specimen was collected from such an index patient for testing at the local laboratory using rapid antigenic point of care tests or polymerase chain reaction (PCR) assays according to local practices, in line with the intention to perform the trial in as much a real life situation as possible. Laboratories were requested to send part of the specimen to the coordination Centre for Infectious Disease Control (CIb) at the Dutch National Institute for Public Health and the Environment (RIVM) for further characterisation and determination of antiviral susceptibility. Upon confirmation of an outbreak by detection of influenza virus in the index ILI patient, study medication was dispatched immediately from a central storage location. Prior to the trial, randomization was performed by an independent statistician, not connected with the study team. The randomization code was used at the central storage facility to prepare blinded study medication in the randomised sequence. The first outbreak received the first blinded package, the second outbreak the second blinded package etc. Outside the central storage facility, no one involved in the study (staff and residents in nursing homes, laboratories, investigators, monitors) were aware of which medication was used, until the end of the study when the code was broken.The unit of randomization was a nursing home unit. Units in which a virological confirmed influenza patient had been identified, were eligible for randomization and randomly allocated to receive either oseltamivir 75 mg or a placebo during 10 days following the diagnosis of the index patient. Index patients received therapeutic oseltamivir 75 mg twice daily for 5 days as per routine practice. All consenting not affected residents in the same unit were offered PEP once daily with the randomised study medication within 48 hours of onset of symptoms in the index patient. Non-consenting residents did not receive any PEP. Units where more than 48 hours had lapsed since start of illness of the index patient were excluded (Figure 1a).

Following the start of PEP, daily monitoring of intake of PEP and of the development of influenza continued for 10 days. Daily information on the intake of PEP, influenza-like illness, pneumonia, additional drug use, admissions and serious adverse events (including death) was collected on a checklist for all consenting participants. Baseline data were collected in a standardised way on demographic characteristics. If influenza-like illness occurred, a nose swab and throat swab were collected for influenza virus detection and further characterisation of detected influenza viruses at the RIVM/CIb. In addition, these participants with ILI were immediately removed from the trial protocol and offered therapeutic oseltamivir. To assess asymptomatic transmission of influenza virus, participants were requested for a nose swab and throat swab for influenza virus detection at the RIVM/CIb at the end of PEP at day 10 irrespective of having symptoms or not. In view of the already existing registration of oseltamivir in this population for this indication, the advanced age and medical condition of most residents, monitoring of adverse events focused on previously reported adverse events (in particular new gastro-enteritis and allergic dermatological events), and on serious adverse events (hospital admissions, deaths). Standard operating procedures were drafted and agreed upon with the participating public health offices, nursing homes and medical laboratories.

### Ethics

Ethical permission was granted by the Utrecht University Medical Ethical Committee. The trial has been registered in the Dutch Trial Register, which is part of the Dutch Cochrane Centre, and which is incorporated in the meta-Register of Controlled Trials, and a unique ISRCTN number (NL27938.041.09) has been allocated. All participants or their legal representative provided written informed consent. The trial was monitored to ensure adherence to Good Clinical Practice.

### Virologic analyses

From all collected specimens received at the central laboratory total nucleic acid was extracted using the MagNA Pure LC or MagNA Pure 96 automated extractor (Roche) and analysed for the presence of RNA of influenza virus types A and B using one-step real-time reverse transcription PCR (rt RT-PCR) on a LightCycler 480 (Roche). Detected type A viruses were further subtyped by rt RT-PCR to determine the heamagglutinin (H) and neuraminidase (N) subtype. Of the detected type B viruses the lineage was determined using rt RT-PCR. All A(H3N2) influenza virus positive specimens were subjected to neuraminidase amino-acid substitution rt RT-PCR to determine the presence of the substitutions E119V and R292K which are the most common substitutions associated with oseltamivir reduced susceptibility in A(H3N2) influenza viruses. If the viral load was high enough (Ct values equal to or less than 30 in the detection rt RT-PCR), the H and N genes of type A and B viruses and the M gene of type A viruses were sequenced directly from the clinical specimen. In addition, these specimens were subjected to virus isolation on tertiary Monkey Kidney (tMK) and Madin Darby Canine Kidney (MDCK) cells, and if successful, the virus isolates were subjected to phenotypic oseltamivir susceptibility testing using the fluorogenic neuraminidase inhibition assay. Detailed protocols and primers and probes information are available on request.

### Outcome

The primary outcome was an influenza virus infection among residents on PEP in a unit with an influenza index case. This was defined as the detection of a new virological confirmed influenza infection in a participant who had developed ILI under study medication in the same unit as the index patient, at least 24 hours after the start of PEP. The secondary outcome was a clinical diagnosis of ILI in participating residents. Unblinding occurred after all data were entered in the database. Analysis was per protocol. The chi-square test was used to test for a treatment effect by comparing the numbers of units with at least one positive outcome in the placebo and oseltamivir groups. The t-test was also used to test for a treatment effect by comparing the proportions of primary and secondary outcomes per unit in the two groups. All analyses were done in the statistical package R.

## Results

**Figure 2:**
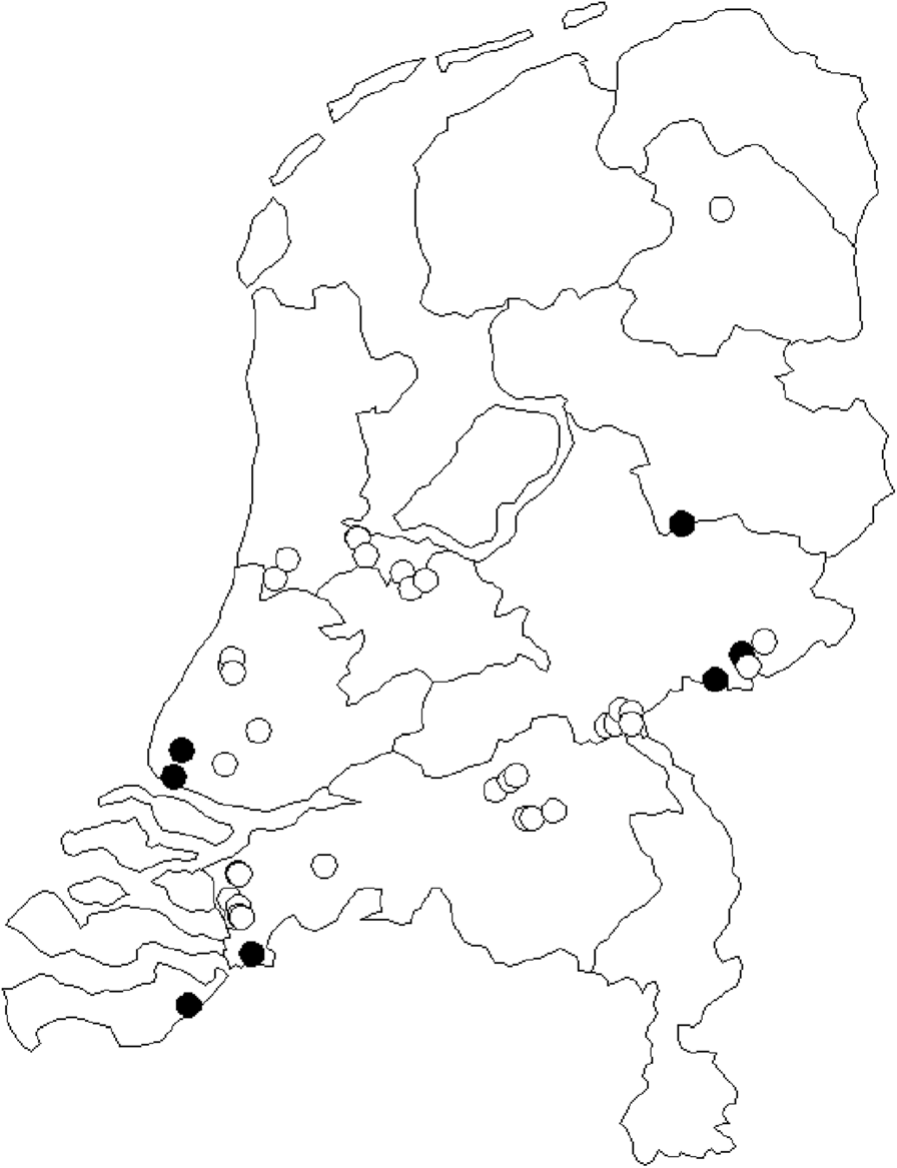
Map of participating 42 homes with outbreak in black.

Forty-two nursing homes were recruited into the trial (see map, Figure 2). During the first two years, immediately following the 2009 pandemic, no outbreaks were reported from any of the participating homes. In 2011/2012, 12 influenza outbreaks were reported, of which 11 were included. In 2012/2013, 10 outbreaks were reported, of which six were included. This is far less than the estimated 60 outbreaks we had expected in 30 nursing homes over a two-year period, based on pre-pandemic data [28]. This may also have contributed to some chance baseline differences between the two groups, but as p-values were small this is not likely. Reasons for non-inclusion of five outbreaks were fewer than five consenting residents on a unit (1x), lack of virological confirmation of influenza in the ILI index case (2x) and/or more than 48 hours having elapsed since the start of the outbreak (2x) (Figure 1a).

In total, 17 outbreaks of influenza in 8 of 42 (19%) nursing homes were recruited into the trial. Fifteen of these outbreaks were caused by influenza virus A(H3N2), for 13/15 the subtype was determined from other ILI cases during PEP as most diagnostic laboratories did not subtype type A influenza viruses. Nearly all of these outbreaks were caused by influenza virus A(H3N2). Two outbreaks (both in the oseltamivir arm) were caused by influenza virus type B. All of the index cases were prescribed therapeutic oseltamivir 75 mg BD for 5 days. In 15 of 17 (88%) outbreaks, in seven homes, PEP study medication was delivered according to protocol, and 99 non-ill residents were included and received PEP. For only one participant, consent was not obtained prior to the start of the influenza season. Of these 15 units, six (36 residents) were allocated to oseltamivir and nine (63 residents) to placebo (see Figure 1 a and b; flow diagrams by units and by residents). Table 1 summarises baseline characteristics of the study population. In the participating nursing home units assigned to placebo, residents were overall slightly younger and less often vaccinated (Table 1).

**Table 1:** Characteristics of study population (n=99 participants)

	**Placebo (n = 63)**	**Oseltamivir (n = 36)**
Units	*9 (60%)*	*6 (40%)*
*Units in first season N (%)*	*6 (67%)*	*3 (50%)*
No. (%) females	*39 (61.9%)*	*26 (72.2%)*
Mean age (years) (SD)	*79.1 (9.4)*	*83.7 (8.4)*
- Female	*81.6 (9.0)*	*85.9 (5.9)*
- Male	*75.0 (8.7)*	*78.0 (11.4)*
No (%) vaccinated		
- 2011/2012	*32 (76.2%)*	*17 (100%)*
- 2012/2013	*19 (90.5%)*	*19 (100%)*
Total (%)	*51 (81.0%)*	*36 (100%)*
Side effects (n participants)	*2*	*5*
Discontinued (n participants)	*2*	*2*

In six of seven units where secondary cases of ILI were diagnosed, specimens from residents with ILI were collected at the start of the ILI and submitted for influenza virus detection; on the 7^th^ unit only an end-of-therapy specimen was available. Influenza virus positive secondary ILI cases were detected in 2/6 (33%) units on oseltamivir (1x influenza virus type B Yamagata lineage, 1x influenza virus A(H3N2)) and in 2/9 (22%) units on placebo (all influenza virus A(H3N2)), which was not a statistically significant difference. Secondary cases of ILI during PEP were diagnosed in 2/6 (33%) units on oseltamivir and in 5/9 (55%) units on placebo, which did not provide statistical evidence of a treatment effect either. Adjusting for size of the unit (taking the fraction of cases as outcome) did not change the lack of statistical significance of either of these estimates (p-values of 0.2 and 0.8 resp.). In 3/9 units on placebo secondary ILI cases could not be confirmed as caused by influenza virus infection, whereas in both units with secondary ILI cases on oseltamivir this was confirmed to be an influenza virus infection. At individual level, 2 (67%) participants with ILI in the oseltamivir group and 6 (67%; 8/9 tested) in the placebo group tested influenza positive. Of all PEP participants tested, regardless of ILI, 3/32 (9%) in the oseltamivir and 10/47 (21%) in the placebo group tested positive.

A(H3N2) positive specimens from index cases (n = 11), secondary ILI cases (n = 6) and specimens collected at end-of-PEP (n = 4) were available for further virological analysis from 1/6 units on oseltamivir and 7/9 units on placebo. None of the sequenced A(H3N2) viruses from the index patients, secondary ILI cases during oseltamivir or placebo PEP, nor the end-of-PEP placebo specimens showed evidence for NA-E119V or NA-R292K or other amino acid substitutions associated with reduced susceptibility for oseltamivir. Four A(H3N2) influenza viruses were available for phenotypic antiviral susceptibility testing; none of them showed reduced inhibition with oseltamivir. The neuraminidase sequences of one index case and of two secondary ILI cases from this unit on placebo PEP were identical and unique in a background of influenza surveillance A(H3N2) viruses from the same time period confirming ongoing transmission of A(H3N2) virus on this unit.

Four participants discontinued the study, of which two died (both in the oseltamivir group) which were considered to be due to advanced age, poor general health and underlying co-morbidities. According to the nursing home physician, these deaths were not related to participation in the trial or study medication. Two of the index cases also passed away in spite of oseltamivir treatment. Otherwise, reported side effects among trial participants were mild and limited. Among 36 residents who took oseltamivir as study medication only a side effect of diarrhoea was reported by two (5.6%) participants. Five of 63 residents on placebo reported side effects: diarrhoea (1.6%), repeated nosebleed (1.6%), sore throat, hoarseness and cough (1.6%) and muscle pain and coughing (3.2%); none of the participating residents discontinued due to side effects.

## Discussion

We failed to find statistically significant evidence for a protective effect of post-exposure prophylaxis with oseltamivir on influenza transmission among exposed nursing home participants. While there was a lower proportion of units where ILI occurred if oseltamivir rather than placebo was used, laboratory-confirmed influenza virus transmission as measured among secondary ILI cases was found in a higher proportion of units allocated to oseltamivir than to placebo. At individual level, an influenza virus infection was detected in fewer residents receiving oseltamivir prophylaxis then in residents who received placebo prophylaxis. Although we had limited data, we also found no evidence that use of oseltamivir as PEP induced antiviral resistance.

The fact that the start of the RCT coincided with the 2009 pandemic was unexpected and the much lower incidence of influenza among the elderly population post-pandemic therefore resulted in an underpowered study: only fifteen nursing home units could be included compared to the desired sample size of 60. Pre-pandemic data suggested that 30% of nursing homes would experience influenza outbreaks each season, on average affecting two units [28]. In our study, no outbreaks were reported in the first two post-pandemic years, and in the additional two years together only 20% of houses reported outbreaks, on average still two units per house being affected. Further extension in time was not possible as the study medication provided would no longer be useable. Even if it had been possible to obtain new study medication similar to the original batches, the low influenza incidence in the winter of 2013/2014 suggests that few additional outbreaks would have been included, not alleviating the lack of power due to attack rates much lower than pre-pandemic. The lower infection rates among elderly populations for influenza virus A(H1N1)pdm09, the 2009 pandemic virus, was already clear shortly after the emergence of the 2009 pandemic [32,33]. Indeed, in none of the nursing home outbreaks in this trial, influenza virus A(H1N1) or A(H1N1)pdm09 was detected, even though these were the dominant influenza strains circulating in the communities during most of these years. Only in the 2011/2012 season, significant circulation of influenza virus A(H3N2) was observed in the Netherlands, coinciding with the year with the largest number of outbreaks [34,35].

In spite of randomisation, some baseline differences were observed among the two groups. Although randomisation is designed to make units comparable with regards to known and unknown confounders, by chance some differences can occur. The lower age in the placebo group might have somewhat reduced the risk of influenza virus transmission occurring in these groups, while the lower vaccination uptake might have resulted in a slightly increased risk. Thus, it is likely that this imbalance between the groups had little overall effect on the outcome of the trial. We did not present adjusted outcomes, as adjustment would contradict the rationale of a RCT, but in any case adjustment did not change the outcomes (data not shown) [36]. Furthermore, in spite of block randomisation by four, a logistical mix-up occurred, resulting in a less optimal randomisation process and unequal group sizes.

Logistical challenges in ensuring that residents would receive PEP within 48 hours of onset were considerable. This did not relate so much to the set-up of the trial (the delay of dispatching the study medication from the central storage was on average less than 2 hours), as to the delay in obtaining informed consent from individual participants, in particular if this needed to be obtained from relatives, as well as delays in the process to confirm influenza virus infection in the index patient (relating to within-house logistics of collecting a sample from a suspected index case, getting the sample to the local laboratory, receiving the virology results, and notifying the responsible professionals). As the effectiveness of oseltamivir is assumed to be strongly correlated with timely administration, this will have reduced our chances of finding an effect if there might have been one. However, as we wanted to assess the real-life effectiveness, we deliberately opted for a real-life situation whereby nursing homes would follow a routine protocol, which would be sustainable outside the trial [37]. Although residents who had not yet consented to participate prior to the outbreak were again approached at the start of the outbreak, this did not result in improved uptake; in this setting rapid decision making could not be ensured. Randomisation should have ensured that deviation from protocol with respect to swabbing, testing and use of the intervention were random. Nevertheless, if start of PEP was relatively late (albeit within 48 hours), this will have resulted in a dilution of any real effect. Indeed, availability of a rapid and reliable bed-side test might be an essential prerequisite to enable timely start of PEP (and therapeutic) oseltamivir in a nursing home setting; rapid start of therapeutic oseltamivir can also limit ongoing transmission.

In recent years, the effectiveness of therapeutic use of oseltamivir has come under scrutiny. In particular, more data have been demanded to assess effectiveness to prevent complications in ill patients [38]. This debate might also have an impact on actual use, as preliminary data from a recent nationwide Dutch study in 34 nursing homes [39] suggested only a minority (less than 20%) of participants with ILI were prescribed therapeutic oseltamivir (J van der Steen/S Hendriks, personal communication), compared to the 89% of nursing home physicians reporting to prescribe therapeutic oseltamivir for ILI in 2006 [28]*.* Also prophylactic use of oseltamivir may need more research in real life situations. As many mathematical models have assumed a significant impact of antiviral use by exposed people on transmission, policy makers have invested in storage of antivirals in case of a severe influenza outbreak. However, model assumptions need to be fed by data, and the need for more data on the actual impact of prophylactic use of antivirals has emerged in discussions on the (cost)effectiveness of prepandemic stockpiling of oseltamivir, following the 2009 pandemic. Our study tried to fill part of this the gap with regards to the need for evidence on the effectiveness of oseltamivir as PEP during the annual outbreaks in nursing homes.

## Conclusions

In spite of an ambitious design, the lack of influenza outbreaks among the elderly in the wake of the pandemic-driven change in dominant circulating influenza strains, has resulted in an underpowered study, which may have contributed to our inability to demonstrate effectiveness of oseltamivir as PEP to reduce the incidence of influenza outbreaks in nursing homes. The debate on the effectiveness of oseltamivir as PEP among nursing home residents remains open therefore and the current recommendation for Dutch nursing homes that if PEP is used, then scientific evaluation of its effectiveness is indicated, still stands. As long as the current low incidence of seasonal influenza in elderly populations persists, it will be challenging to achieve the power needed in a real life situation in a single study to demonstrate effectiveness or lack of effectiveness in a conclusive manner, and meta-analysis of single studies might be needed. Well-designed trials such as this one provide the much needed data to enable such integrated assessments. At the same time, insight into trends in the burden of disease of influenza remains essential, as the logistical challenges needed to ensure timely implementation are considerable, and can only be justified if recurrent outbreaks continue to cause significant morbidity and mortality.

## Abbreviations

CIb: Dutch centre for infectious disease control (CIb) located at the RIVM; ILI: Influenza-like illness; PCR: Polymerase chain reaction; PEP: Post-exposition prophylaxis; RCT: Randomised controlled trial; RIVM: Dutch national institute for public health and the environment.

## Competing interests

The authors declare that they have no competing interest.

## Authors contributions

MvdS designed the study, contributed to the implementation, was responsible for the interpretation of the data and drafted the paper. AM was responsible for the virological analysis and supervision, contributed to the interpretation of the data and the drafting of the paper. FK oversaw the implementation of the trial, contributed to the analysis and the drafting of the paper. RE contributed to design, implementation of the study and the drafting of the paper. HC contributed to design, interpretation of the data and the drafting of the paper. PO contributed to the virological analysis and contributed to the drafting of the paper. JF was responsible for the statistical analysis of the data and contributed to the drafting of the paper. M-J V supervised the implementation, contributed to the implementation, analysis and drafting of the paper. All authors read and approved the final manuscript.
